# Orbital Floor Fractures: Treatment and Diagnostics – A Survey Among Swiss, German and Austrian Maxillofacial Units

**DOI:** 10.1177/19433875241245498

**Published:** 2024-04-18

**Authors:** Thomas Burger, Kathleen Fan, Johannes Brokmeier, Florian M. Thieringer, Britt-Isabelle Berg

**Affiliations:** 1 Department of Oral and Cranio-Maxillofacial Surgery, 30262 University Hospital Basel, Basel, Switzerland; 2 Faculty of Medicine, University of Basel, Basel, Switzerland; 3 Oral and Maxillofacial Surgery Department, 4616 King’s College Hospital NHS Foundation Trust, London, UK; 4 Alumni, 9144 Ruprecht Karls University Heidelberg, Heidelberg, Germany; 5 Department of Biomedical Engineering, Swiss MAM Research Group, University of Basel, Allschwil, Switzerland

**Keywords:** orbital floor fracture, blow-out fracture, orbital fracture, orbital reconstruction, patient specific implant, printed anatomical model

## Abstract

**Study Design:**

N/A.

**Objective:**

This study investigated the different ways of orbital floor reconstruction with special focus on reconstruction materials, imaging modalities (intra-/ post-operative), 3D printing and navigation.

**Methods:**

The heads of all governmental-run or associated cranio-maxillofacial surgery units in Switzerland, Austria and Germany were asked in person or received an email link for an online survey with 12 questions.

**Results:**

The return rate was 57%. The most often selected number of reconstructions was between 10 and 50 per year. Resorbable polydioxanone (PDS) foils (41%) and titanium mesh (18 %) were most often used to reconstruct the orbital floor. 31% use 3D Navigation intraoperative. Post-operative imaging was most often performed with CBCT (34.5%) in patients without complications, whereas CT scans were most often performed (63.3%) in patients with persisting complications. In total, 27% stated that they never use preformed orbital plates, and the remaining units use them more or less regularly. 48% have access to a 3D printer and 75% of the respondents use patient specific implants.

**Conclusions:**

The majority of the participating units prefer to use resorbable material for the reconstruction of the orbital floor defects. 3D printing facilities are not available in the majority of units, but it can be expected that the number of units with 3D printing facilities will rise in the near future.

## Introduction

Orbital floor fractures are frequent occurring fractures of the mid-face. Common causes are, for example, falls, assaults, sports accidents or traffic accidents.^1-4^ In all cases, a specific external force is applied to the area of the orbit or lateral mid-face. Despite its relatively protected internal position, the orbital floor represents a weak point susceptible to fracture due to its low bone wall thickness of .37–.59 mm.^5^ The treatment of an orbital floor fracture can be conservative or by means of surgical reconstruction. The indication for surgery depends on the symptoms and the severity of the fracture. If surgical reconstruction of the orbital floor is indicated, there are a number of different procedures and reconstructive materials to choose from. Resorbable and non-resorbable materials as well as alloplastic or autologous ones are available.^1^ Depending on a variety of factors, publications of reconstruction with titanium mesh, Medpore (porous absorbable polyethylene), PDS foil or autologous bone are well described.^6^ Apart from the materials utilized for reconstruction, the planning of the procedure pre-operatively is essential. Techniques of 3D printed anatomical models or patients’ specific implants (PSI) are precise and time saving operating procedures.^7,8^

Intra-operatively, monitoring of the reconstruction can be challenging due to a restricted view.^8^ Therefore, intra-/post-operative radiological examination is essential. C-arm, digital computed tomography (CBCT) or computed tomography (CT) are devices available intra-/post-operatively, depending on the unit’s preference and the available equipment. If there is a non-favourable surgical outcome, for example, motion restriction or persisting double vision, further imaging such as magnetic resonance tomography (MRI) might be recommended.^9^

Due to the variety of options to diagnose, perform and follow-up orbital fracture, this study aims to assess the current situation of surgical procedures for orbital floor reconstruction and diagnostics, most commonly performed in Swiss, German and Austrian maxillofacial surgery units.

## Materials and Methods

**Figure 1.**
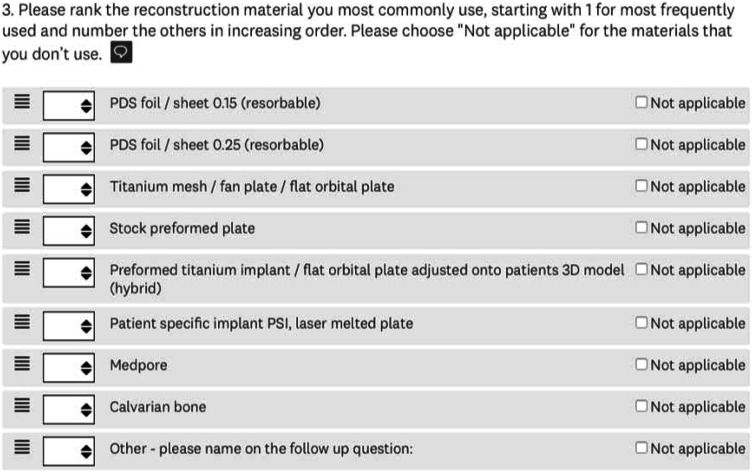
Example of question of the online survey questionnaire.

A questionnaire with 12 questions regarding treatments and diagnostics of orbital floor fractures was developed. The questionnaire was transferred to the online survey platform ‘Surveymonkey’. The complete questionnaire can be seen in the online appendix. An example of a question is shown in Figure 1.

In Switzerland, 5 out of 7 questionnaires were handed out in person and the answers were added to the online survey. For all the other participants, a link created by ‘Surveymonkey’ was emailed to the chief physicians of all known university and state hospitals in Switzerland, Austria and Germany. A reminder email was sent via the chief physician’s secretary office 5 months later. The email addresses were taken from the homepages of the hospitals. In the case of no public email addresses present, contact via an online contact form was used. The list of hospitals with a maxillofacial unit was taken from the directories of the Swiss Society for Oral and Maxillofacial Surgery (SGMKG) and the corresponding societies in Austria (ÖGMKG) and Germany (DGMKG). A total of 98 hospitals were found appropriate and included in the survey, in detail 7 units in Switzerland, 10 in Austria and 81 in Germany. The participants were able to skip individual answers in case they didn’t want to answer all of them.

The descriptive statistic was performed using Excel (Version 14.6.9) the other statistical tests were performed using the software ‘R’.^10^ For the statistics, an alpha level of 5% was used for all tests (p = .05). Differences between countries regarding average OFF reconstructions were tested with the chi square test. Zero inflated Poisson regression models were used to compare material usage between countries. Binomial tests were used to test whether some options were picked above a 50% chance.

This study was conducted according to the principles of the Declaration of Helsinki. Since no patients were involved and the questionnaire could be filled out anonymously, an ethical approval by the local ethics committee (Ethical Committee North West/Central Switzerland) was not required.

## Results

The response rate was 57% (56 out of 98 possible units), including 7 out of 7 from Switzerland (100%), 7 out of 10 from Austria (70%) and 42 out of 81 from Germany (52%). The following part presents the answers to the individual questions in the questionnaire. Since the participants were able to skip individual answers, missing data was marked with ‘not applicable’. Likewise, multiple answers were possible in some questions, so the cumulative percentages for these questions could exceed 100%.

The first question assessed how many orbital floor fractures were reconstructed by each unit on average per year using alloplastic material or calvarian bone. The majority stated that they reconstruct between 10 and 50 fractures (48%) per year, followed by 50–100 fractures (30%). Other possible answers: fewer than 10 (7%), 100–150 (9%), 150–200 (2%) and >200 (4%).

The second question was about the frequently used reconstruction materials. There is no clear order of popularity (see Table 1).

**Table 1.** Ranking of the Reconstruction Material Most Commonly Used, Starting With 1 for Most Frequently Used and Numbering the Others in Increasing Order. ‘Not Applicable’ was Used for the Materials That Were Not Used by the Participants. Percentages Measured Against 56 Total Answered Questionnaires.

Answer	1	2	3	4	5	6	7	8	9	Not Applicable	Total Answers
PDS foil .15	13	2	4	2	1	0	0	0	1	21	46
	23.2%	3.6%	7.1%	3.6%	1.8%				1.8%	37.5%	
PDS foil .25	10	10	2	1	2	0	0	0	0	14	39
	17.8%	17.8%	3.6%	1.8%	3.6%					25.0%	
Titanium mesh	10	12	6	7	0	1	0	0	0	11	47
	17.8%	21.4%	10.7%	12.5%		1.8%				19.6%	
Stock preformed plate	1	7	9	1	2	0	0	0	0	23	43
	1.8%	12.5%	16.1%	1.8%	3.6%					41.0%	
Preformed titanium implant	3	5	6	7	4	3	0	0	0	18	46
	5.4%	8.9%	10.7%	12.5%	7.1%	5.4%				32.1%	
Patient specific implant PSI	6	8	9	8	7	0	0	1	0	11	50
	10.7%	14.2%	16.1%	14.2%	12.5%			1.8%		19.6%	
Medpore	1	1	1	0	2	1	0	0	1	34	41
	1.8%	1.8%	1.8%		3.6%	1.8%			1.8%	60.7%	
Calvarian bone	0	0	3	3	3	2	2	0	0	30	43
			5.4%	5.4%	5.4%	3.6%	3.6%			53.6%	
Other: (Specified on the next question)	12	3	2	3	0	0	1	1	0	22	44
	21.4%	5.4%	3.6%	5.4%			1.8%	1.8%		39.2%	

Among the most frequently used materials are the PDS films .15 mm and .25 mm, and the titanium mesh. The titanium implants, the patient specific implant PSI and the stock preformed plate are also used by many clinics but not ranked as number one. Calculating all rankings together 75% of the participating units already use PSIs. Medpore sheets and autologous bone from the calvaria are used by only a few clinics. The participants were also able to write in other materials in a follow-up question. Answers such as Ethisorb Patch (14 times), Bio Gide Collagen Membran (3x), Neuro Patch (Polyester-urethane dura substitute) (2x) and an Antral Balloon (1x) were mentioned. A PDS sheet and the preformed plate were also mentioned in this section here, although these possibilities were already part of the selectable answers to the previous question.

**Figure 2.**
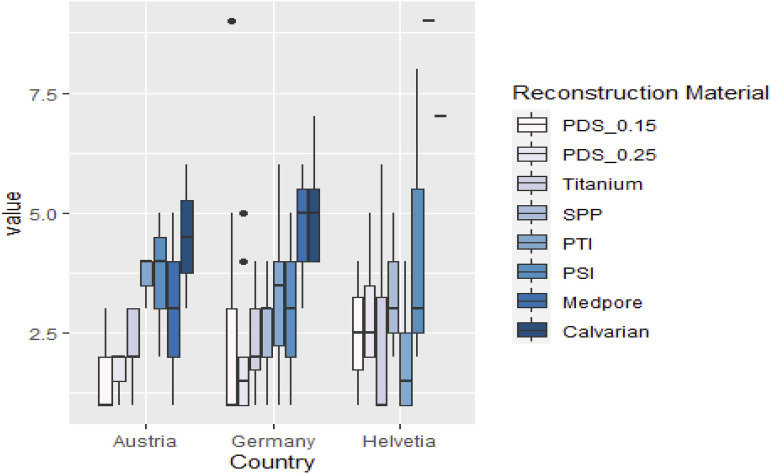
Visualization of the distribution of the reconstruction material.

Regarding the distribution of the reconstruction material in Germany, Austria and Switzerland, the distribution is visualized in Figure 2.

In the next section, control of the reconstruction intra-operatively was inquired. The responses revealed that the majority of respondents (67%) do not use intraoperative imaging. Intraoperative navigation was used in 31% of the respondents. 15% use of C-arm imaging and 9% acquire an intraoperative cone beam computed tomography (CBCT) scan. The percentages exceed 100% due to the possibility of multiple answers. Navigation and additionally C-arm or CBCT are used by some respondents.

The following frequencies were stated for intra-operative control: 48% do not use intraoperative imaging at all. An additional 10.7% of respondents do not use radiological intraoperative imaging but use 3D navigation when using preformed materials for reconstruction. Single-digit percentages of respondents each indicated that they rarely (1.7%), sometimes (7.1%) or often (3.6%) use intraoperative radiological imaging. 8.9% of respondents always use radiological imaging.

When intra-operative imaging was performed: 32% stated that they do not do post-operative imaging if they have already done intra-operative imaging. 5.3% of the respondents take an additional post-operative CBCT after intra-operative imaging, and 1.7% of the respondents take an additional post-operative conventional radiograph. The majority, 61% of respondents indicated that they do not use intraoperative imaging and therefore the question does not apply to them.

**Figure 3.**
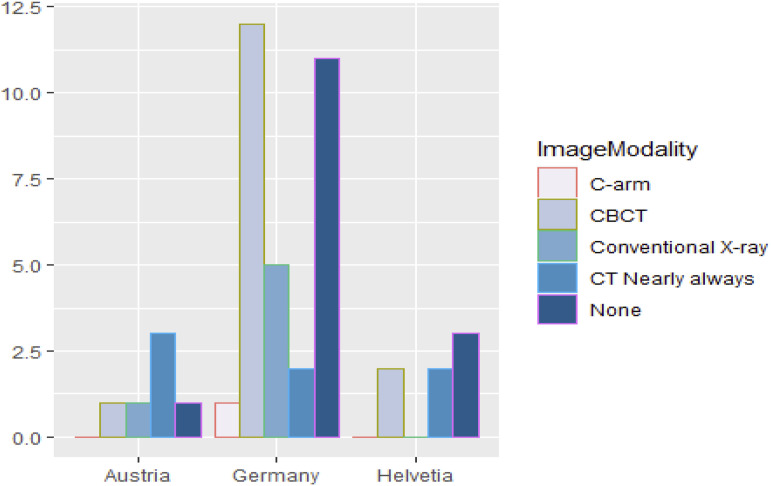
Image modalities used for the orbital floor reconstruction in a patient without complications by country. The bars indicate absolute numbers.

In patients without complications, 36% of the participants perform a CBCT post-operatively, while 30% don’t perform any post-operative imaging at all. Post-operative computed tomography (CT) or conventional X-ray is each used by 16% of the respondents and C-arm imaging is used post-operatively by a single respondent (1.8%) in Germany. MRI or ultrasound is not used by any of the respondents. When the data is assessed regarding the countries, following distribution is visible (see Figure 3).

In patients with double vision after orbital floor reconstruction, the used image modalities are displayed in Table 2. In case more than one modality was favoured, the methods were numbered, starting with ‘1’ for the method most frequently by the participant and ‘not applicable’ for the ones which were not used.

**Table 2.** Imaging Modalities Used in Patients With Double Vision After Orbital Floor Reconstruction. In Case More Than One Modality was Favoured, the Methods Were Numbered, Starting With 1 for the Method Most Frequently by the Participant and ‘Not Applicable’ for the Ones Which Were Not Used.

Answer	1	2	3	4	Total Answers
CT	31	13	1	0	50
	55.4%	23.2%	1.8%		
CBCT	14	11	4	0	38
	25.0%	19.6%	7.1%		
MRI	6	17	13	0	44
	10.7%	30.4%	23.2%		
C-arm	0	0	0	0	31
Ultrasound	0	0	0	0	30
Conventional X-ray	0	1	1	1	31
		1.8%	1.8%	1.8%	
Not applicable	2	1	1	0	31
	3.6%	1.8%	1.8%		

The use of printed 3D models of the orbit to adapt a mesh or orbital plate pre- or intra-operatively was asked. 35% of respondents indicated that they do not use this method. 22% of the respondents send the CT data externally as they do not have a printer available, 26% of respondents did not send CT data externally because they have a 3D printer/model printer available and 17% of the respondents have a printer available but do send the data externally in exceptional cases.

In terms of frequency of use, 18.5% of respondents use 3D models often, but not always. 20.4% of respondents use them sometimes and 37.0% rarely use them. 24.1% of the respondents never use 3D models of the orbit. No one stated that they always use 3D models.

Concerning PSI custom laser-melted implants, 27% of the respondents do not use PSI at all. 36% use it rarely, 28% use it sometimes and 15% of the respondents use it often. Only 3.6% of the respondents always use PSI for reconstruction the orbital floor.

No significant differences could be found between countries regarding average reconstructions or used materials, tested by the chi square test and inflated Poisson regression models, respectively.

## Discussion

Orbital floor fractures are among the most common fractures of the facial bones.^11^ Surgical reconstruction will be performed in large defects or in patients with clinical symptoms such as enophthalmos or eye motility restrictions. Depending on the hospital’s equipment and preferences of the performing surgeon different ways of reconstructing the orbital floor are performed. The present study aimed to assess which modalities, reconstruction materials and imaging methods are frequently used.

To reach a conclusive number of answers, an online survey was conducted among all known Swiss, Austrian and German maxillofacial units who perform this kind of surgery. Our response rate of 57% was in a satisfying range. The response rates in web-based surveys vary strongly.^12-15^ Due to the simple, impersonal way to of an email with a link, online surveys with reply rates as low as 13.5% are published.^16^ A published study by Feveile et al. didn’t show a major difference between the response rate comparing mailed vs telephone surveys. Interestingly they showed that the ‘don’t know’ option was more often opted by participants taking part in the mailing survey in comparison of participants of the telephone survey.^17^ Compared to surveys mailed by post, which often include a pre-paid return envelope, emailed surveys cost less and can be conducted faster. With many affordable telephone flat rates, the same applies to surveys conducted by telephone. Postal response rates also vary widely and don’t always receive high participation rates.^12^ Shih and Fan^13^ compared response rates from web or mail surveys and found that the response rate also varied depending on the group of participants.

In the present study, the question from which minimum defect size the orbital floor needs to be reconstructed was not assessed. A publication by Kunz et al. suggested that defects <3 cm don’t need reconstruction,^18^ and the question about the best timing for reconstructions was not posed. Various publications cover this aspect.^19-21^ One focus of this study was to find out preferences concerning reconstruction materials. The choice of which material used depended on the size and location of the defect as well as the surgeon’s preference. The size and scope of the hypothetical defect in the survey was purposely not described in detail, so as to not influence the respondents’ answers towards any reconstruction materials. The idea was to capture the generally preferred reconstruction materials. PDS foil (either .15 mm or .25 mm) is the No. 1 choice by the biggest share of the participants (41%), followed by titanium mesh (18%) and PSIs (11%).

PDS (Polydioxanone, Ethicon GmbH, Norderstedt DE) is a resorbable material, available in different thicknesses (.15 mm, .25 mm and 0.5 mm). This material will over time be replaced by scar tissue. This can take up to 180 days and might still leave a defect.^22^ The foil is easy to handle but due to its lack of radiopacity and is almost not visible in a CT scan.

In comparison to PDS foil, titanium mesh and PSI made from titanium are clearly visible in the CT scan and easily distinguishable from possible artifacts. The positional stability is an advantage of the titanium mesh in comparison to the PDS foil especially in larger defects. One possible disadvantage of titanium mesh, as Holtmann et al. have shown, are some patients reporting foreign body sensations and cold feeling in the area of reconstruction.^21^ This study show 18% of units bend and cut the titanium mesh free-hand, other units use pre-printed 3D models, with the healthy side mirrored over the fractured side for pre-bending titanium meshes either pre-operatively or intra-operatively. Models produced by printing technologies are very accurate.^23^ However, 43% of the participants have a 3D printer available. Differences among the countries were detectable. In Germany 35.7%, in Austria 42.8% and in Switzerland 85.7% of the units had their own 3D printer available. Reasons for this difference are difficult to detect. In Switzerland only two hospitals aren`t university hospitals therefore research including 3D printing might be of more importance. No unit stated that they always use 3D printed models to preform plates. It is expected that utilization of this technique will increase due to the advantages of 3D models. Using 3D printed models and pre-bending the titanium mesh saves operating room time^7,24^ and therefore saves cost. 75% use patient specific implants (PSI) and 15% stated that they use them often. Wilkat et al. conclude that reconstructions in complex fractures might benefit from PSIs because of a higher accuracy with ‘selective laser-melted PSIs’ in comparison to PDS foils.^25^ Timoshchuk et al. point out that when comparing PSIs and preformed implants, no differences concerning complications was assessable.^26^

In this study, navigation was used by 31% of the participants. Intraoperative navigation can reduce errors regarding PSI position and consequently reduce complications.^27^ From Probst et al.’s point of view, it is not necessary to use navigational systems for inserting PSIs because the accuracy is sufficient enough to place the implant free-hand.^28^ From an educational perspective, however, it shouldn’t be forgotten that there might be situations in which a 3D model or PSI are not readily available, and where surgeons should still be able to reconstruct an orbital floor fracture without the help of these tools.

In regard to imaging, the majority of participants (67%) don’t use intraoperative imaging at all. 15% of the participants have C-arm imaging available and 9% can acquire a intraoperative cone beam computed tomography (CBCT) scan. For those units who wish to have intra-operative imaging available, a possibility would be to find out if co-operations with the neuro-orthopaedic or spine surgery are possible. In these departments, the use of a C-arm is more common.

In patients without complications, 36% of the participants will still perform a CBCT post-operatively, but 30% won’t perform any post-operative imaging at all. Post-operative computed tomography (CT) or conventional X-ray is less often used by the participants (16% each). Due to the respective size difference of the three countries and therefore the number of maxillofacial surgical units, statistics concerning differences between countries are very inconclusive.

A different approach is chosen in patients with persisting double vision or other symptoms: The majority will perform a CT scan (55%) or a CBCT scan (25%). 11% of the participants would choose a MRI scan as first choice. Post-operative motility disorders can be challenging and detections of causes leading towards this complication can be difficult to capture. Since soft tissues are difficult to judge in a CBCT scan, in persisting motility disorders, a CT scan should be given priority or even an oculo-dynamic MRI scan should be considered. In an oculo-dynamic MRI, movement of the eyes can be detected in near-real time helping to see adhesions or palsies.^9^

## Conclusion

The reconstruction methods of orbital floor fractures in Switzerland, Austria and Germany still vary from unit to unit. When the size of the defect is not taken into account, the majority of the participating units prefer to use resorbable material for the reconstruction of the orbital floor defects. Through the last decade, 3D technology gained has more importance. Although 3D printing facilities are not available in the majority of units yet, it can be expected that the number of units with 3D printing facilities will rise in the near future.^29^

## Data Availability

The raw data presented in this study are available on request from the corresponding author.*
